# Coriander response to nitrogen fertilizer sources in different competing levels of weeds

**DOI:** 10.1016/j.heliyon.2024.e26816

**Published:** 2024-02-22

**Authors:** Fatemeh Alaei, Saeideh Maleki Farahani, Hassan Habibi, Mohammad Hossein Fotokian, Mostafa Khodadadi

**Affiliations:** a Department of Crop Production and Plant Breeding, College of Agriculture, Shahed University, P. O. Box 18155/159, Tehran, Iran; b Seed and Plant Improvement Institute. Karaj, Iran

**Keywords:** Fatty acid, N fertilizer, Oil content, Seed yield, Weed competitive

## Abstract

The competition of weeds with crops and nutrient management has a significant effect on the yield and economic efficiency of a country. This study aimed to evaluate the impacts of sulfur-coated urea and common urea on the yield and fatty acid composition of three coriander genotypes (Nahavandi, Pishgam, Ethiopia) under weeded and unweeded conditions. Traits including 1000 seeds weight, fruit yield, content of oil, and composition of fatty acids were examined. Nitrogen fertilizer and weeding treatments significantly improved the weight of 1000 seeds and weeds decreased the yield of fruit. The highest fruit yield was obtained by the Ethiopia genotype in weed-free conditions. Results showed that N fertilizer increased the oil percentage of coriander fruit. Urea fertilizer resulted in the highest oil content in the Nahavandi and Pishgam genotypes under weeded plots in the first and second years, respectively. Also, petroselinic, linoleic, and palmitic acids were the major coriander fatty acid composition. Nahavandi genotype showed the highest palmitic acid. Also, urea in the weed condition led to increase the linoleic acid content in the Nahavandi genotype. Overall, results showed that N fertilizer, especially urea, improved the quality characteristics of coriander fruits.

## Highlights

• *Coriander* Genotypes are important sources of oil.
• Weed competition reduced the yield and oil of coriander.
• N improved *Coriander* Genotypes performance in weeded plots.
• Pishgam genotype had more oil content under different climatic conditions and management practices.

## Abbreviations

SCU: Sulfur Coated Urea
CRU: Controlled-Release Urea
NUE: Nitrogen-Use Efficiency

## 1 Introduction

Medicinal plants are cultivated to obtain corresponding amounts of secondary metabolites, which determine the quality and final value of the harvested medicinal plants [1]. Therefore, management strategies to keep them free of chemical residues are very important. Coriandrum sativum L. is an herbaceous annual crop, belonging to the family of Apiaceae [2]. It is native to the Mediterranean and is grown worldwide including Iran. The seeds and leaves of these species are a suitable source of essential oils and have widely applied in cosmetics, foods, and pharmaceutical industries [3]. Furthermore, coriander fruit contains 9.9–27.7% of fatty oils. These fatty oils have anti-inflammatory and anti-aging activites. The most abundant fatty acid in coriander oil is petroselinic acid (65.7–76.6 %). It was reported that it can be broken down to produce lauric acid, as a raw material for softeners, and adipic acids, used in the synthesis of nylon polymer [4]. Coriander is a spice crop which has a special place in food flavoring industries. In addition to fatty oils and essential oils, coriander fruits contain 21.5% carbohydrate, 32.5% fiber, 14 % protein, 11.1% moisture, and 4.3% of minerals. Also, coriander leaves contain significant amounts of A and C vitamins [5]. Regarding the importance and value of the coriander metabolites, inefficient chemical fertilizers, especially nitrogen fertilizers are one of the major challenges in agricultural industries. Nitrogen is an essential nutrient for crop growth, and has been used in large quantities. The most common type of nitrogen fertilizer used worldwide is urea. Although, in comparison with other types of nitrogen sources, urea has the greatest nitrogen (46%) content [6], its high water solubility could leach the N content of urea into the surrounding soils before it get absorbed by the plants. In addition to leaching caused by rain and irrigation, N can also be lost due to the evaporation of ammonia and decomposition of urea. Furthermore, it has been reported that about 55% of urea fertilizers do not get absorbed by treated plants [7]. One of the effective methods to improve the yield of crops is the increasing application of nitrogen fertilizer. However, extreme nitrogen fertilization has resulted in low nitrogen use efficiency. The over-application of nitrogen to arable lands may have adverse environmental effects through runoff, leaching, and N-based gaseous emissions. Also, low NUEs lead to lower income for growers. An appropriate source, level, and fertilizer time is needed for efficient N management. This guarantees that a sufficient amount of nitrogen is useable for the crops and results in the maximization of the crop's yield and NUEs. Besides, it helps in minimizing the negative effect of nitrogen on the environment. It was observed that split nitrogen application can raise fruit yields and nutrient use efficiency, but additional fertilization costs can make the method uneconomic [8]. One possible approach to reduce the nitrogen losses from the surface-applied urea is the urea granule coating. The coating material can be composed of sulfur, urease inhibitor (agrotain), and other biodegradable materials. Controlled-release urea is designed to match the nitrogen release rate with the crop's uptake rate [9]. In addition, the use of Control Release Urea (CRU) as a basis can be less time-consuming compared to conventional nitrogen fertilizers, which require split fertilization [10]. Developing a management strategy is very important to improve nitrogen use efficiency for coriander [11]. One of the effective methods to solve this problem is to use Slow-Release Fertilizers (SRF) such as Sulfur-Coated Urea (SCU) is a type of fertilizer that is coated in sulfur, which slows down its release of nitrogen into the soil. SCU is a sulfur-coated fertilizer that regulates nitrogen release into soil, ensuring optimal plant growth and yield. Nitrogen release gradually could reduce leaching, volatilization, and losses of denitrification. Evidence suggests that SCU plays an effective role, especially in irrigated coarse-textured soils [12]. The yields of rice and canola were 6–8.1% and 6.2–15.4 % higher in the CRU. Also, Zheng et al. (2016) found that CRU raised the performance of wheat and maize compared to conventional urea [9]. The average increase in NUE and yearly net profit of slow-release fertilizer compared to urea were 15.4–38.4% and 16–20.8%, respectively. It is reported that a 30% reduction in the N rate is possible with CRU while, by maintaining the performance and protecting the soil, labor/time is saved [13]. It has been shown that CRU has improved NUE in several systems of production. However, environmental conditions strongly affect the effectiveness of CRU [7]. Weed competition is another challenge that has a major impact on plant yield. They are considered a major issue for almost all products [1]. The competition of weeds with crops for nutrients, water, space, and solar radiation leads to a 20–50% yield reduction [14]. Today, however, the role of weeds is recognized in maintaining the stability of agricultural ecosystems. There are different methods to remove weeds. One of the cheap and sustainable strategies for weed management is the use of competing varieties [15].

Limited information is available regarding the impact of various N fertilizer sources on the coriander yield and physiological processes, especially when encountering weed interference. The purpose of this research was to investigate the potential of utilizing SCU and common urea under weed competition to determine optimum fertilization and weed management for chemical-free coriander. Ensuring the quality and safety of production is necessary not only for humans but also for the environment in which we live, which has caused this issue to attract the attention of researchers and producers [16].

## 2 Material and methods

### 2.1 Experimental design and plant materials

**Fig. 1:**
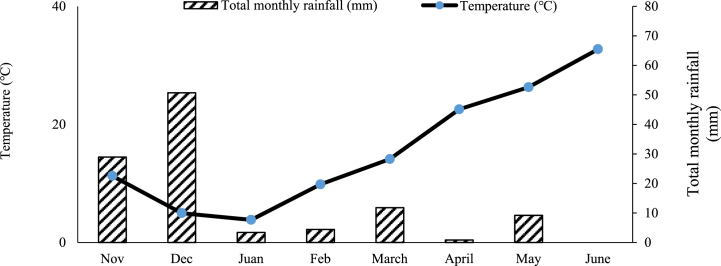
Total monthly precipitation and mean monthly air temperature at experimental site between Now 2020 and June 2021.

Plant materials were three coriander genotypes of Nahavandi, Pishgam, and Ethiopia. The field studies were performed at Shahed University, Tehran, Iran (35o34' E, 51o8' N, altitude: 1191 m Altitude) in November 2020 and March 2021. The climate of the experiment site was subtropical with a yearly mean rainfall of 251 mm. The changes in precipitation and mean temperature of air are presented monthly in Fig. 1. To determine the physical and chemical character of the soil, a sample of soil from a 0–30 cm depth of the surface layer of the soil was taken (Table 1). A factorial experiment based on a randomized complete block design with three replicates was applied in both years. An experimental treatment consisted of three factors including: 1) nitrogen fertilizer sources (Control treatment: without nitrogen fertilizer; SCU: with rate of N, 70 kg/ha, and urea: with rate of N, 70 kg/ha), 2) genotypes (Nahavandi, Pishgam and, Ethiopia), and 3) weeding (weedy and weeding by hand). Nahavandi and Ethiopian genotypes were obtained from the Karaj Seed and Plant Improvement Institute in Iran, and the Pishgam genotype was obtained from the Ganjineh Bazr Sabz Fanavar Asia seed company. The experimental plot size was 2 × 2 m2 dimensions with a plant spacing of 25 cm × 10 cm. A space of 1.5 m between plots and 2 m between blocks was maintained. The urea (46% N) was applied two times: once before planting (60% of the total urea) and again at the vegetative stage of coriander when plant height was 10–15 cm (40% of the total urea). The SCU fertilizer (31% N, the release longevity was 45 days) was applied once before planting as basal fertilizer. This fertilizer was obtained from the Kian Arjan Company in Iran. The application depth of basal fertilizer was 10 cm. Before irrigation, Top dressing urea was broadcasted to the soil surface. Also related to weeded plots, weeding was done manually as soon as the weed appeared, during the season of growth.

**Table 1:** Physical and chemical characteristics of study field soil.

Year	Depth	Texture	Sp (%)	EC Ds/m	pH	O.C (%)	Total N (%)	P (ppm)	K (ppm)
2020–2021	0–30	Loamy	36	3.36	7.94	1.76	0.15	51.8	753.9
2021–2022	0–30	Loamy	25	2.4	7.94	1.3	0.11	55.1	874.4

Sp: Soil porosity, EC: electrical conductivity, pH: potential of hydrogen, O.C: organic carbon.

### 2.2 Sampling and measurement

#### 2.2.1 Fruit yield

At the final of the growth period, coriander plants of 1 m2 per plot were harvested, and fruit yield per plot was measured. The harvest of the first and second cultivation of Pishgam and Ethiopian genotypes was done on May 19th and June 16th, 2021 respectively. The same was done for Nahavandi on May 29th and June 23rd, 2021.

#### 2.2.2 Fatty acid extraction

##### 2.2.2.1 Content of oil

The hexane solution was used in a soxhlet-type apparatus (ACS grade, Reag. Ph Eur., ≥ 99 %; manufactured by Merck company, Germany) to extract the oil content. Then 150 mL of solvent was spilled into the soxhlet-type device, and 10 g of grounded fruit from each treatment was added to the solvent. The prepared solvent was boiled (10 h), and after concentration, the oil was obtained by removing the solvent [17].

##### 2.2.2.2 Composition of fatty acids

The composition of fatty acids was identified by methylation and gas chromatography [18]. 0.10 g of the sample of oil was poured into a 5 ml test tube, then 3 ml, heptane and 2 ml, 0.01 M sodium hydroxide solution, and shaken at a speed of 10,000 rpm for 15 s. Eventually, a 1-μL sample was prepared, and this prepared sample was then injected into the gas chromatography apparatus. The analysis of fatty acid methyl esters was done by an Agilent 7890A GC (Agilent Technologies, Inc. 2010) that has a ﬂame ionization detector (FID), using a BPX capillary (part number 054980) column, and N as a carrier gas (Agilent Technologies, Inc. 2010). The primary temperature of the column was regulated at 165 °C and after 10 min, a rise of temperature from 165 °C to 200 °C at 1.5 °C per minute was programmed. 250 °C and 280 °C were set for injector and detector temperature, respectively [19].

### 2.3 Statistical analysis

Normality of residuals distribution and equality of residuals variance were through shapiro-wilk normality test and levene's test for homogeneity of variance, respectively. A combined analysis of variance using F-test and $y_{\text{ijklm}}=\mu +Y_{j}+iY_{\text{ij}}+G_{k}+F_{l}+W_{m}+YG_{\text{jk}}+YF_{\text{jl}}+YW_{\text{jm}}+GF_{\text{kl}}+FW_{\text{lm}}+GW_{\text{km}}+\text{YG}F_{\text{jkl}}+\text{YG}W_{\text{jkm}}+\text{YF}W_{\text{jlm}}+\text{GF}W_{\text{klm}}+\text{YGF}W_{\text{jklm}}+\varepsilon_{\text{ijklm}}$.

linear model was applied to evaluate the efficiency of the factors explored. Where μ, $Y_{j}$, $G_{k}$, $F_{l}$, $W_{m}$, $iY_{\text{ij}}$, and $\varepsilon_{\text{ijklm}}$ are total mean, year, genotype, fertilizer, weed, replication within year effects and experimental error, respectively. Comparison of means were done using the Duncan's multiple range test at P < 0.05. Data analysis were done using SPSS v.26 software.

## 3 Results

The pooled analysis of variance showed that the interaction of genotype, weed, and year significantly affected the seed yield and the interaction of fertilizer, and weed, which significantly affected the 1000 seeds weight (Table 2). The effect of the interaction of genotype, fertilizer, and weed also significantly influenced the seed oil content in two years (Table 3). Related to the fatty acid composition, the combined variance analysis showed that genotype and weed had significant effects on palmitic acid and palmitoleic acid respectively. Also, the interaction of fertilizer and genotype had a significant effect on myristic acid, palmitoleic acid, and stearic acid and the interaction of genotype, fertilizer, and weed significantly affected the α Linoleic (Table 4).

**Table 2:** The combined analysis of variance for the effect of year, fertilizer, genotype, and weed on plant growth parameters.

Source of variation	df		Seed 1000 wt			Seed yield	
		MS	F value	P value	MS	F value	P value
Year (Y)	1	49.229	3.479	0.311	12272.605	0.132	0.796
Block (Year)	4	0.918	0.471	0.757	6895.551	0.868	0.488
Genotype(G)	2	163.809	60.834	0.016	23549.210	0.227	0.815
Fertilizer(F)	2	1.742	4.522	0.181	196915.264	7.257	0.121
Weed(W)	1	152.574	11.001	0.186	1992585.998	31.822	0.112
Y × G	2	2.693	3.371	0.519	103621.185	1.554	0.396
Y × F	2	0.385	0.332	0.783	27133.888	0.863	0.545
Y × W	1	13.869	84.647	0.892	62617.183	0.682	0.469
Y × G × F	4	1.612	1.140	0.451	6456.246	0.895	0.542
Y × G × W	2	0.603	0.425	0.680	67422.236	9.336	0.030
Y × F × W	2	0.965	0.681	0.556	32201.282	4.461	0.095
Y × G × F × W	4	1.414	0.725	0.578	7215.449	0.909	0.464
G × F	4	0.419	0.260	0.890	7167.401	1.110	0.461
F × W	2	22.897	23.722	0.040	150975.110	4.688	0.176
G × W	2	1.410	2.338	0.300	19665.269	0.292	0.774
G × F × W	4	4.989	3.528	0.125	10478.892	1.452	0.363
Error	68	1.951			7940.990		

df: degree of freedom. Y: sowing date in 2020 and 2021.

**Table 3:** The variance analysis to the effect of genotype, fertilizer, and weed on plant oil content (autumn 2020 and spring 2021).

Source of variation	df	Oil autumn 2020			Oil spring 2021		
		MS	F value	P value	MS	F value	P value
repetition	2	0.05	0.33	0.71	0.16	2.317	0.11
Genotype(G)	2	8.85	51.59	0.00	1.29	18.287	0.00
Fertilizer(F)	2	4.24	24.71	0.00	7.77	109.635	0.00
Weed(W)	1	241.25	1405.72	0.00	29.30	412.998	0.00
G × F	4	1.72	10.02	0.00	2.07	29.287	0.00
F × W	2	5.53	32.24	0.00	0.80	11.378	0.00
G × W	2	5.01	29.21	0.00	0.76	10.800	0.00
G × F × W	4	1.74	10.17	0.00	0.18	2.593	0.049
Error	34	0.17			0.07		

df: degree of freedom.

**Table 4:** The variance analysis to the effect of year, genotype, fertilizer, and weed on fatty acid composition.

Source of variation	df	C14:0		C16:0		C16:1		C18:0		C18:1		C18:2		C18:3		C20:0	
		MS	P value	MS	P value	MS	P value	MS	P value	MS	P value	MS	P value	MS	P value	MS	P value
Year (Y)	1	0.078	0.11	4.75	0.268	0.095	0.271	0.251	0.386	74.45	0.126	14.30	0.224	0.125	0.377	0.001	0.847
Block (Year)	2	0.011	0.00	1.89	0.000	0.033	0.000	0.114	0.003	1.707	0.469	1.252	0.023	0.095	0.000	0.013	0.010
Genotype (G)	2	0.003	0.261	1.28	0.024	0.009	0.725	0.03	0.780	4.84	0.352	0.176	0.251	0.04	0.256	0.001	0.765
Fertilizer (F)	2	0.002	0.260	0.023	0.844	0.005	0.401	0.003	0.915	1.92	0.452	0.37	0.650	0.004	0.267	0.005	0.275
Weed (W)	1	0.002	0.127	0.205	0.669	0.013	0.028	0.26	0.283	9.46	0.435	2.28	0.510	0.004	0.637	0.00	0.075
Y × G	2	0.001	0.545	0.031	0.839	0.024	0.999	0.107	0.115	2.63	0.940	0.059	0.916	0.016	0.182	0.003	0.918
Y × F	2	0.001	0.454	0.127	0.586	0.003	0.747	0.029	0.236	1.58	0.745	0.68	0.373	0.001	0.859	0.002	0.485
Y × W	1	0.00	0.784	0.62	0.208	2.45	0.967	0.06	0.174	6.27	0.100	2.44	0.195	0.01	0.381	5.01	0.983
Y × G × F	4	5.41	0.896	0.07	0.203	0.001	0.916	0.004	0.476	2.08	0.524	0.17	0.402	0.003	0.013	0.002	0.799
Y × G × W	2	0.001	0.078	0.12	0.101	0.003	0.514	0.013	0.120	0.34	0.863	0.6	0.095	0.004	0.09	0.003	0.540
Y × F × W	2	5.43	0.790	0.15	0.076	0.011	0.174	0.008	0.220	0.83	0.710	0.37	0.171	0.007	0.003	0.002	0.675
Y × G × F × W	4	0.00	0.878	0.02	0.894	0.004	0.272	0.003	0.930	2.22	0.417	0.13	0.769	0.00	0.985	0.004	0.221
G × F	4	0.00	0.031	0.03	0.730	0.006	0.038	0.026	0.042	1.91	0.532	0.24	0.376	0.001	0.722	0.002	0.470
F × W	2	0.001	0.06	0.13	0.527	0.002	0.832	0.009	0.473	0.35	0.699	0.39	0.492	0.00	0.975	0.003	0.357
G × W	2	0.001	0.664	0.33	0.273	0.008	0.282	0.017	0.430	5.54	0.058	0.46	0.566	0.002	0.607	0.001	0.691
G × F × W	4	0.00	0.471	0.06	0.248	0.002	0.741	0.011	0.145	1.91	0.555	0.11	0.574	0.003	0.012	0.001	0.848
Error	34	0.001		0.1		0.003		0.1		2.2		0.29		0.002		0.003	

df: degree of freedom. Y: sowing date in 2020 and 2021. C14:0; Myristic acid, C16:0; Palmitic acid, C16:1; Palmitoleic acid, C18:0; Stearic acid, C18:1; petroselinic, C18:2; Linoleic acid, C18:3; *α*-Linolenic acid, C20:0; Arachidic acid.

### 3.1 Growth and yield

**Fig. 2:**
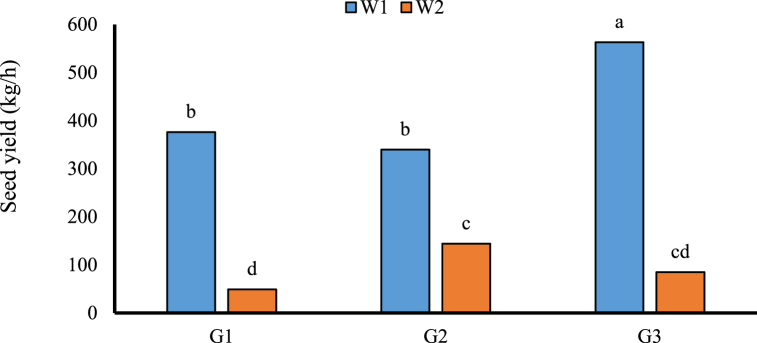
The interaction effect of genotypes and weed on seed yield in autumn cultivating season. Different letters in columns indicate significant differences between treatments at (*p* < 0.05) based on Duncan's test. W1: Weeded; W2: Un weeded; G1: Nahavandi; G2: Pishgam; G3: Ethiopia.

**Fig. 3:**
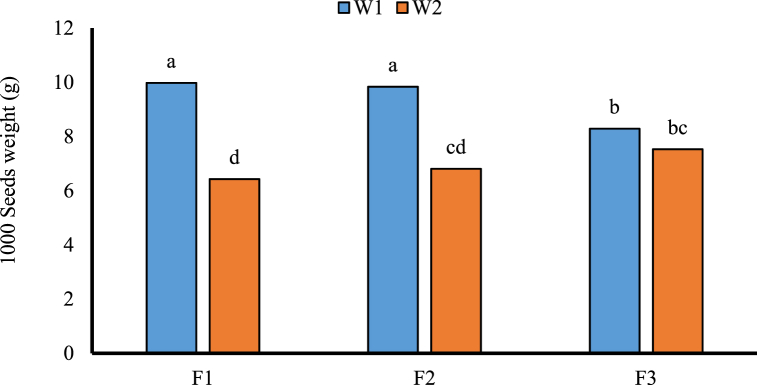
The effect of N fertilizer and weed on 1000 seeds weight. Different letters in columns indicate significant differences between treatments at (*p* < 0.05) based on Duncan's test. F1: Urea; F2: Sulfur coated urea; F3: Control; W1: Weeded; W2: Un weeded. The data presented is the average of the both autumn and spring seasons.

The weed competition have decreased the fruit yield of all genotypes. The highest fruit yield was exhibited by Ethiopia genotypes at weeded plots in the first cultivation (Fig. 2) but that was insignificant in the second cultivation. Also, results showed that overall N fertilizer significantly increased the 1000 seeds weight in weeded plots although in un-weeded plots, fertilizer supply has a detrimental effect on the 1000 seeds weight (Fig. 3).

### 3.2 Oil content and composition of fatty acids

**Fig. 4:**
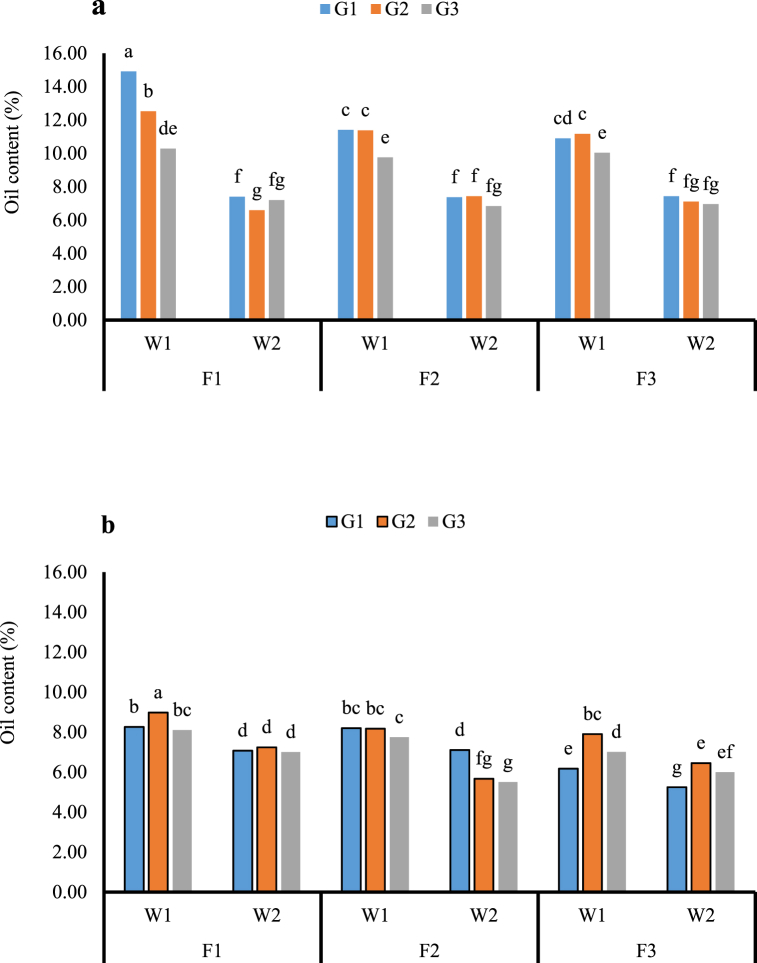
The impact of N and weed competition on oil content of three coriander genotypes in two seasons (a: autumn cultivating; b: spring cultivating). Different letters in columns indicate significant differences between treatments at (*p* < 0.05) based on Duncan's test. F1: Urea; F2: Sulfur coated urea; F3: Control; W1: Weeded; W2: Un weeded; G1: Nahavandi; G2: Pishgam; G3: Ethiopia.

In case of the oil content, due to the non-uniformity of variances for oil percentage, a combined analysis of variance was not performed and the data have analyzed separately for each year. All studied factors significantly affect oil content. The oil content of the first cultivation (autumn) was significantly higher than the second cultivation (spring) especially in the weeded plots of all genotypes, although under low temperatures at sowing, Nahavandi followed by the Pishgam genotype produced more oil content (Fig. 4 a), but cultivation under higher temperatures which caused shortening growing period, Pishgam had more oil content (Fig. 4 b). The content of oil in the three genotypes declined by weed competition in both cultivation. The nitrogen fertilizer improved the content of oil compared with the non-nitrogen-fertilizer treatment in weeded plots. The oil content of Nahavandi and Pishgam genotypes was the highest in urea fertilizer and weed-free treatments in the first and second cultivation, respectively (Fig. 4 a and b).

**Fig. 5:**
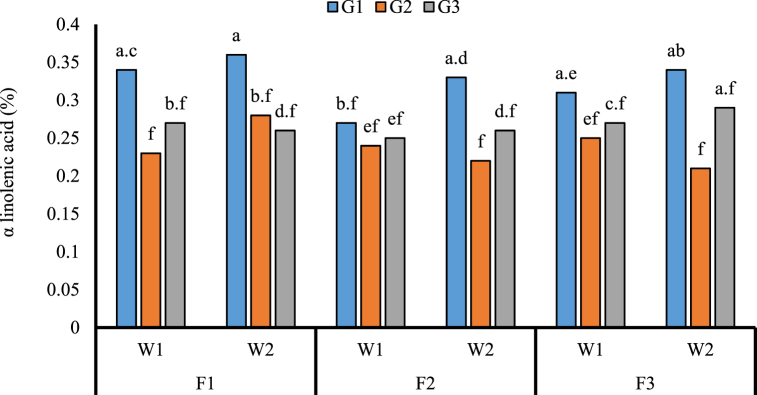
The impact of N and weed competitive on α linolenic acid content of three coriander genotype. Different letters in columns indicate significant differences between treatments at (*p* < 0.05) based on Duncan's test. F1: Urea; F2: Sulfur coated urea; F3: Control; W1: Weeded; W2: Un weeded; G1: Nahavandi; G2: Pishgam; G3: Ethiopia.

Fatty acids compositions in the selected genotypes were palmitic acid, myristic acid, stearic acid, palmitoleic acid, petroselinic acid, linolenic acid, and arachidic acid (Table 4). The palmitoleic acid content increased in the un-weeded plot and the Nahavandi genotype showed the highest palmitic acid content (Table 5). These genotypes showed different responses to N fertilizer for fatty acid components. Myristic, palmitoleic, and, stearic acid content increased under sulfur-coated urea, urea, and control fertilizer in the Nahavandi and Ethiopian, respectively (Table 6). Also, αlinoleic acid content was high in the Nahavandi genotype under un-weeded and urea-treated plot (Fig. 5).

**Table 5:** The mean comparison for fatty acid values affected by fertilizer, weed and genotype using the Duncan's multiple range test.

Treatments		C16:0 (%)	C16:1 (%)
Genotype	Nahavandi	4.15a	0.37a
	Pishgam	3.70b	0.34a
	Ethiopia	3.83b	0.37a
Fertilizer	Urea	3.93a	0.37a
	Sulfur coated urea	3.88a	0.36a
	control	3.87a	0.34a
Weed	weeded	3.88a	0.35b
	Un weeded	3.94a	0.38a

The same letters per column indicate non-significant differences at *p* < 0.05. C16:0; Palmitic acid, C16:1; Palmitoleic acid.

**Table 6:** Interaction effect of genotype and fertilizer on composition of fatty acids.

Treatments				
Genotype	Fertilizer	C14.0 (%)	C16.1 (%)	C18.0 (%)
Nahavandi	Urea	0.11 ab	0.36 abc	1.00 ab
	sulfur coated urea	0.13 a	0.34 abc	0.95 b
	Control	0.11 ab	0.38 abc	0.97 ab
Pishgam	Urea	0.09 ab	0.35 abc	1.05 ab
	Sulfur coated urea	0.12 ab	0.33 BCE	1.08 ab
	control	0.09 b	0.33 BCE	0.98 ab
Ethiopia	Urea	0.11 ab	0.41 a	1.03 ab
	Urea coated urea	0.103 ab	0.38 abc	1.04 ab
	control	0.09 b	0.32 c	1.14 a

The same letters per column indicate non-significant differences at *p* < 0.05. C14:0; Myristic acid, C16:1; Palmitoleic acid, C18:0; Stearic acid.

## 4 Discussion

Nitrogen fertilizers significantly caused an improvement in the fruits yield of coriander in the weeded plots. Similarly, Yeganehpoor. et al. (2016) [20] and Abdelkader. et al. (2018) [21] showed that the vegetative features of coriander improve with nitrogen supplement. The increase in vegetative growth is due to the positive impact of N in the synthesis of amino acids, chlorophyll, and stimulating the cells growth [22]. In un-weeded plots, the impact of weed infestation was more dominant than N fertilization. Therefore, different sources of nitrogen had no significant difference in the fruits yield. Control release urea (CRU) enables the nutrients to be released gradually and according to the plant nutrient demand during the growth period. Through the application of CRF, the efficiency of nutrient use increases, which ultimately increases the quality and productivity of the product [23]. However, some studies showed U and CRU treatments did not show a significant difference in the seed yield [24]. Furthermore, in the Taihu region of China, the rice fruit yield in the CRU treatment was lower than U [25]. Izgi (2020) did not find any significant effect of N use on fruit yield. In Izgi’ trial, the impact of different N sources was not significant for fruit yield. Differences in genotypes, soil, and environmental conditions can cause different test results [26]. Also, reports have shown that plants show different reactions to different doses of nitrogen [13,27]. Also, results showed that overall N fertilizer significantly increased the 1000 seeds weight in weeded plots although in un-weeded plots, fertilizer supply has a detrimental effect on the 1000 seeds weight. Indeed, as indicated in reports, weeds are nitrogen-demanding [14]. In our experiment, the Ability to withstand competition (AWC) of the control treatment (without fertilizer) significantly exceeded that of urea and sulfur-coated urea treatments. This aligns with the observed higher seed weight in the control under weed competition, emphasizing the impact of nitrogen fertilization. A key agronomic determinant factor for coriander fruit yield is weed control [28]. Coriander grows slowly in the early vegetative stages and is very sensitive to the competition of weeds in this stage [29]. Higher grain yield in weeded plots is due to the loss of weed competition and high access to water, nutrients, and sunlight [14]. It has been reported that coriander yield is more dependent on weeding compared to N Fertilization. This hypothesis has thought to be more observable for higher nitrogen rates while the different genotypes in presence of different weeds and environmental conditions, showing different reactions. One of the most important quantitative traits which evaluated in this experiment is the seed yield. The seed yield in this experiment, has been affected by the interaction effects of genotype, weed, and cultivation season. As the results show, the fruit yield of all genotypes was significantly reduced in the weed competition condition in the first cultivation. However, the effect of genotype on weeds in the second cultivation was not significant. It may be due to the presence of different types of competing weeds at different sowing dates, though it can affect the competition capacity of coriander with the competitor weeds. Similar responses were obtained on the oil content as the other significant parameter in coriander cultivation. It has clearly shown that coriander genotypes are more susceptible to weeds growth in the winter or in lower temperatures. In the first cultivation, the dominant competing weeds were the Grameneae family with fast vegetative growth, which strongly affected the growth of the coriander. But, in the second cultivation, the dominant weeds were the Amaranthacea family, which had a slower initial vegetative growth than the coriander, so the plants were suitably established with minor competition damage. In this regard, reports have been presented considering the effects of weed germination time on the crops yield [30]. Also, it was probably due to the coriander's inhibitory allelopathy effects on these weeds. According to the reports, the inhibiting role of Apiaceae family plants on Amaranthus retroflexus, which is one of the dominant weeds in the spring season, has been confirmed [31]. In this regard, further detailed experiments and additional targeted tests are needed. Phytochemicals are described as a defense system in the plants. Petroselinic acid is the main component of coriander's fruits fatty acid. It composes more than 70 % of the total fatty acid content of the Apiaceae's fruits [32]. Oil of coriander fruit is approved as a safe and healthy foodstuff when permissible doses of 60 mg per day are used and it has been used in different industries. Several reports have shown the environmental effects on the quantity and quality of the coriander oil. For example, post-transcriptional regulation during the synthesis of fatty acids depends on temperature, which can ultimately influence the composition of the fatty acids [33].

In weeded plots, nitrogen, especially urea fertilizer, significantly increased the coriander seed oil content as reported in prior research [34]. The growth characteristics and production of plants can be affected by nutrients [35]. N is one of the major nutrients needed by crops. Nitrogen involves in various organic compounds production and, provides energy for crops to grow and develop. Therefore, providing a sufficient amount of nitrogen fertilizer can enhance the quality and quantity of the medicinal metabolites in the cultivated plants [36]. Cell wall elasticity has a vital role in the division process of the plant cells. It has been reported that an increase in the NO3^−^ level results in the higher osmotic pressure and causes inflation pressure that improves the cell division process and consequently improves the morphological characteristics of the plant cells. It has been shown that the medicinal and phytochemical compounds of Echinacea purpure increase significantly with the increase of NO3− level, which is due to the improvement in the growth of Rhizomata cumradicibus [37].

It has been seen that weed infestation significantly reduces the percentage of seed oil. The existence of annual weeds during the growing season of coriander could lead to severe loss in seed and oil yield. During the weed infestation, a decline in the seed and oil yield occurs simultaneously with a decrease in the growth traits [38]. Weed-competing situations influences the function of the enzymes involved in the fatty acid biosynthesis. For example, the Amaranthus palmeri L. shading effect on Glycin max L. led to a change in seed fatty acid composition [39].

Unlike the weeded plots where urea fertilizer significantly increased the percentage of oil, in the unweeded plots, the effect of fertilizer treatment in two growing seasons showed different results. In the autumn cultivation season, nitrogen has an insignificant effect on the weed competition plots which indicates that weeds were a more limiting factor than nitrogen fertilizers. But in the spring cultivation season, the effect of nitrogen fertilizer was significant and it has increased the percentage of oil in un-weeded plots. This could be because of the presence of different types and amounts of the weeds in two cultivation seasons. In the present study, different fatty acid compounds showed different reactions to the different treatments. Palmitic acid content was influenced by the genotypic effect and Nahavandi genotype showed the highest value. Izgi (2020) reported the impact of genetics on some fatty acid compounds of coriander is stronger than the effect of nitrogen [26]. Two-way interaction including genotype and, fertilizer had significant effect on production of myristic acid, palmitoleic acid, and stearic acid. The Nahavandi genotype treated with urea fertilizer with sulfur coating showed the highest myristic acid percentage, the Ethiopian genotype treated with urea fertilizer showed the highest palmitoleic acid, and the Ethiopian genotype without applying fertilizer (control) showed the highest stearic acid. Nitrogen and sulfur are key elements of qualitative features of the oily plants. Since nitrogen could be removed from the soil due to high leaching, oxidation, reduction, nitrification, etc., using management approaches such as utilizing slow-release fertilizers could help to overcome this problem because it could synchronize the fertilizer release and plant nutrient demands. For this reason, utilizing the urea with sulfur coat as a slow-release fertilizer, leads to increase in chlorophyll content, photosynthesis capacity, canopy growth, and plants yield and quality. Therefore, availability of the nitrogen during the plants demand, along with the presence of sulfur, could be an efficient solution [40]. Weeds are biotic stresses and can influence the quantitative and qualitative properties of the plants due to the competitions they can create for different nutrients and resources within the crops [35]. Higher oil content in the first cultivation (2020) compare to the second cultivation (2021) in the weed-free plots shows that lower temperature during the growth season stimulates assimilation of nutrients and oil production increment. Although competition with the weeds probably reduces the oil production, as was seen in treatments with weed competition, the oil content was lower across all genotypes and fertilizing treatments in both years. Then it can be concluded that it is possible to increase the coriander's oil content significantly by weeding and sowing at lower temperatures during growth season. In this regard, it is necessary to study the effects of different weed species on the growth and yield of the coriander genotypes. Palmitoleic acid content increased in plots with weed competition conditions. In various reports, the effect of non-biotic stresses such as drought stress, temperature, etc. on fatty acids production has been reported, which affects the fatty acid structure by affecting the functions of chloroplast and its enzymes [41]. Also, one of the unsaturated fatty acids found in coriander fruit is α-linoleic acid, whose genotype, nitrogen, and weed factors significantly affect its α-linoleic acid content. The highest percentage of this compound belonged to the Nahavandi genotype treated with urea under the weeds competition condition. There is limited data about the impact of different weeds on the fatty acid composition of plants, but it seems that weeds are causing the competition situation for plant nutrients and light resources and resulting in abiotic stresses that affect the fatty acid structure in the plants. In this regard, the effect of adverse environmental conditions on the content of oil and fatty acid compounds observed in Lallemantia iberica L. and Lallemantia royleana L. [42], in Fabaceae and Asteraceae families plants [43] and in coriander [44]. By considering the purpose of cultivation (quantitative, qualitative yield), it is possible to decide on the selection of genotypes, the appropriate source of fertilizers, and even the time of cultivation. According to the results obtained from this experiment, due to the reduction of the damage caused to the yield of coriander, in the conditions of competition with spring season weeds, it is possible to pay special attention to the cultivation time to reduce the use of herbicides. Overall, according to the different reactions of coriander genotypes to nitrogen fertilizer sources and weeds during two growing seasons, it is possible to choose the appropriate treatment according to the purpose of cultivation.

## 5 Conclusion

The present study highlights that N fertilizer and weeding inﬂuenced the crop growth properties, fruit yield, and oil content of three coriander species. In this regard, employing a proper management method such as using sulfur-coated urea fertilizers with a control release system can meet the plant needs and reduce the environmental pollution to some extent. Also, data related to the impacts of N fertilization on interactions between the competition of weeds and crops may help to develop weed management approaches. Weeding increases the oil and seed yield of three coriander genotypes under low and high temperatures during the growth period. We found that the coriander is more sensitive to weed competition than fertilization during growth period, although we found genetic variety in weed competition. Thus, it is necessary to focus on weed competition capacity weeding timeframe, and weed control methods to improve the crops qualitative and quantitative yield. From an environmental and, economical point of view, it is suggested to sow coriander early in the spring without weeding and using sulfur-coated urea fertilizers to reduce the cost of labor, fertilization and weeding and avoid environmental pollution by using less herbicide, urea fertilizer.

## Funding

Funding for this study was provided by 10.13039/501100011696Shahed University, Tehran, Iran.

## CRediT authorship contribution statement

**Fatemeh Alaei:** Writing – original draft, Visualization, Validation, Methodology, Investigation, Formal analysis, Data curation, Conceptualization. **Saeideh Maleki Farahani:** Writing – review & editing, Visualization, Supervision, Resources, Project administration, Methodology, Investigation, Funding acquisition, Data curation, Conceptualization. **Hassan Habibi:** Writing – review & editing, Methodology, Conceptualization. **Mohammad Hossein Fotokian:** Writing – review & editing, Validation, Software. **Mostafa Khodadadi:** Writing – review & editing, Validation, Software.

## Declaration of competing interest

The authors declare that they have no known competing financial interests or personal relationships that could have appeared to influence the work reported in this paper.
